# Classical Signaling and Trans-Signaling Pathways Stimulated by *Megalobrama amblycephala* IL-6 and IL-6R

**DOI:** 10.3390/ijms23042019

**Published:** 2022-02-11

**Authors:** Jixiu Wang, Qianhui Sun, Jian Zhang, Huanling Wang, Hong Liu

**Affiliations:** 1 Key Lab of Freshwater Animal Breeding, Ministry of Agriculture and Rural Affair/Key Lab of Agricultural Animal Genetics, Breeding and Reproduction of Ministry of Education, College of Fisheries, Huazhong Agricultural University, Wuhan 430070, China; wangjixiu@webmail.hzau.edu.cn (J.W.); sunqianhui@webmail.hzau.edu.cn (Q.S.); a715561702@163.com (J.Z.); hbauwhl@hotmail.com (H.W.); 2 Engineering Research Center of Green Development for Conventional Aquatic Biological Industry in the Yangtze River Economic Belt, Ministry of Education, Wuhan 430070, China

**Keywords:** teleost, IL-6, soluble IL-6R, classical signaling, trans-signaling, JAK2/STAT3, MEK/ERK

## Abstract

Interleukin-6 (IL-6) is a multipotent cytokine. IL-6 plays a dual role in inflammation through both classical signaling (IL-6 binds membrane IL-6 receptor/IL-6R) and trans-signaling (IL-6 binds soluble IL-6R). However, the regulation of IL-6 activity, especially the regulation of signaling pathways and downstream genes mediated by IL-6 trans-signaling, remains largely unclear in teleost. Grass carp (*Ctenopharyngodon idellus*) hepatic (L8824) cells, kidney (CIK) cells, and primary hepatocytes were used as test models in this study. First, the biological activity of recombinant blunt snout bream (*Megalobrama amblycephala*) IL-6 (rmaIL-6) and sIL-6R (rmasIL-6R) was verified by quantitative PCR (qPCR) and western blot. The western blot results showed that rmaIL-6 significantly upregulated signal transducer and activator of transcription 3 (STAT3) phosphorylation in L8824 cells and primary hepatocytes, while rmaIL-6 in combination with rmasIL-6R (rmaIL-6+rmasIL-6R) significantly upregulated STAT3 phosphorylation in all types of cells. Furthermore, maIL-6 and maIL-6+rmasIL-6R could only induce extracellular-signal-regulated kinase 1/2 (ERK1/2) phosphorylation in L8824 cells and CIK cells, respectively. Therefore, IL-6 mainly acts by activating the janus kinase (JAK)/STAT3 pathway rather than the mitogen-activated protein kinase (MEK)/ERK pathway. Finally, the activation of the JAK2/STAT3 pathway was shown to be essential for the generation of *socs3a* and *socs3b* induced by IL-6 trans-signaling after treatment by JAK2/STAT3 pathway inhibitors (c188-9 and TG101348). These findings provide functional insights into IL-6 classical signaling and trans-signaling regulatory mechanisms in teleost, enriching our knowledge of fish immunology.

## 1. Introduction

Interleukin-6 (IL-6) was first identified in humans as B cell stimulatory factor 2 (BSF-2), which induces B cells to produce immunoglobulin (Ig) [1]. IL-6 is produced by several cell types including monocytes/macrophages, endothelial cells, adipocytes, and hematopoietic cells and acts on a wide range of cell types such as T/B cells, macrophages, hepatic cells, vascular endothelial cells, and hematopoietic stem cells [1,2,3,4,5,6,7]. As a typical cytokine, IL-6 has pleiotropic effects in inflammation, immune response, and hematopoiesis [8,9]. 

When IL-6 sends signals into cells, it binds to a heterodimeric receptor complex consisting of a high-affinity chain interleukin-6 receptor (IL-6R) and glycoprotein 130 (gp130), and then triggers two major signaling pathways: the extracellular-signal-regulated kinase (ERK)/mitogen-activated protein kinase (MEK) pathway and the Janus kinases (JAK)/signal transducer and activator of transcription 3 (STAT3) pathway [10]. The inflammatory cytokine IL-6 upregulates antimicrobial peptides (*hamp*) expression by activating the IL-6R/JAK2/STAT3 pathway [11]. STAT3 regulates its own endogenous inhibitor, SOCS3, forming a negative feedback loop [12]. In rats, IL-6 induces the activation of the STAT3/SOCS3 pathway in the liver, but not other downstream pathways such as STAT1, ERK1/2, and PI3K [13]. In human cells, the JAK2/STAT3 signaling pathway has been shown to promote IL-6 expression [14,15].

The proinflammatory and anti-inflammatory effects of IL-6 appear to originate from its capacity to activate multiple signaling pathways in a cell type-specific manner [16,17]. For classical signaling, IL-6 binds to membrane-bound IL-6R (mIL-6R) and activates intracellular signaling cascades via gp130. IL-6 classical signaling is primarily limited to hepatocytes and immune cells (macrophages and certain other leukocyte populations), which express IL-6R on their surface [18,19]. Thus, the number of cells targeted by IL-6 classical signaling is restricted. The body also produces a soluble IL-6 receptor (sIL-6R) that is released into the circulation after proteolytic cleavage of the mIL-6R protein or following translation from alternatively spliced mRNA [20,21]. Signaling by IL-6 in combination with sIL-6R is called trans-signaling. Due to the uniform distribution of gp130, this excitatory IL-6 and sIL-6R complex can, in principle, activate all cells [20]. The two different signaling events have divergent functions. Classical signaling is associated with regenerative and anti-inflammatory functions, while trans-signaling is linked to pro-inflammatory functions [22,23,24,25].

Inhibitors have been widely used in the study of signaling pathways and in clinical medicine. c188-9, a small-molecule inhibitor of STAT3, targets the phosphotyrosyl peptide binding site within the STAT3 SH2 domain and does not inhibit upstream JAK or Src kinases [26]. c188-9 has been used to inhibit the phosphorylation of STAT3 and the proliferation of cancer cells [27,28]. On the other hand, Fedratinib (TG101348) is a selective JAK2 inhibitor that is indicated for the treatment of adults with intermediate-2 or high-risk primary or secondary myelofibrosis [29].

The *il-6* gene has been identified in several teleost, such as zebrafish (*Danio rerio*, Hamilton 1822) [30], fugu (*Takifugu rubripes*, Temminck & Schlegel 1850) [31], *Paralichthys olivaceus* (Temminck & Schlegel, 1846) [32], *Sparus aurata* (Linnaeus, 1758) [33], *Larimichthys crocea* (Richardson, 1846) [34], rainbow trout (*Oncorhynchus mykiss*, Walbaum 1792) [35], and blunt snout bream (*Megalobrama amblycephala*, Yi Bolu 1955) [36]. In teleost, IL-6 has been proved to play vital roles in the immune response, inducing the expression of *hamp* and regulating cytokines genes (*il-1β* and *il-6*) and *socs1–3* [33,37,38]. Additionally, in fugu, IL-6R was cloned for the first time, confirming that IL-6 and sIL-6R activated STAT3 phosphorylation [39]. In grass carp, sIL-6R can participate in the immunoregulatory function of IL-6, thereby further upregulating the expression of *socs3* [40]. In Nile tilapia (*Oreochromis niloticus*, Linnaeus 1758), sIL-6R could combine with IL-6 to promote the upregulation of *il-1β* and *il-6* and has an agonistic effect in IL-6-mediated inflammation [41]. Due to its pronounced immunoregulatory effects, IL-6 and sIL-6R might be essential for the initiation of immune defenses in fish. However, little is known about IL-6 classical signaling and trans-signaling transduction mechanisms in fish.

The blunt snout bream *il-6* gene could be strongly induced in *Aeromonas hydrophila* (Kluyver & Van Niel, 1936) in many tissues, indicating that *il-6* plays an active role in the defense against pathogens [36,42]. IL-6 amino acid sequence in blunt snout bream (maIL-6) shares 91% identity with that of grass carp (*Ctenopharyngodon idellus*, Cuvier et Valenciennes 1844) (ciIL-6), but only 23–30% identity with those of mammals, birds, and amphibians [36]. Therefore, to explore the regulation mechanisms of IL-6 classical signaling and trans-signaling in fish, we first verified whether L8824 cells (grass carp hepatocyte cells with IL-6R) can receive signals from recombinant IL-6 and whether recombinant maIL-6 (rmaIL-6) has biological activity. Whereafter, the activation of the JAK/STAT3 and MEK/ERK pathways in L8824 cells, CIK cells (grass carp kidney cells without IL-6R) [40], and primary hepatocytes of grass carp was investigated. Additionally, whether rmaIL-6+rmasIL-6R regulates the expression of downstream genes (*il-6*, *hamp*, *socs3a*, and *socs3b*) through the JAK2/STAT3 pathway was verified.

## 2. Results

### 2.1. Sequence Analysis of maIL-6 and masIL-6R

As shown in Supplementary Figure S1A, the cDNA sequence of *mail-6* is 1045 bp and contains an open reading frame (ORF) of 699 bp, encoding a 232 aa protein with a signal peptide of 24 aa. The full-length cDNA of *mail-6r* is 3739 bp, with an ORF of 1797 bp, encoding 598 aa. The signal peptide region of maIL-6R is 1–21 aa-long, the extracellular region comprises 1–491 aa, the transmembrane region 492–514 aa, and the intracellular region 515–598 aa (Supplementary Figure S1B).

**Figure 1:**
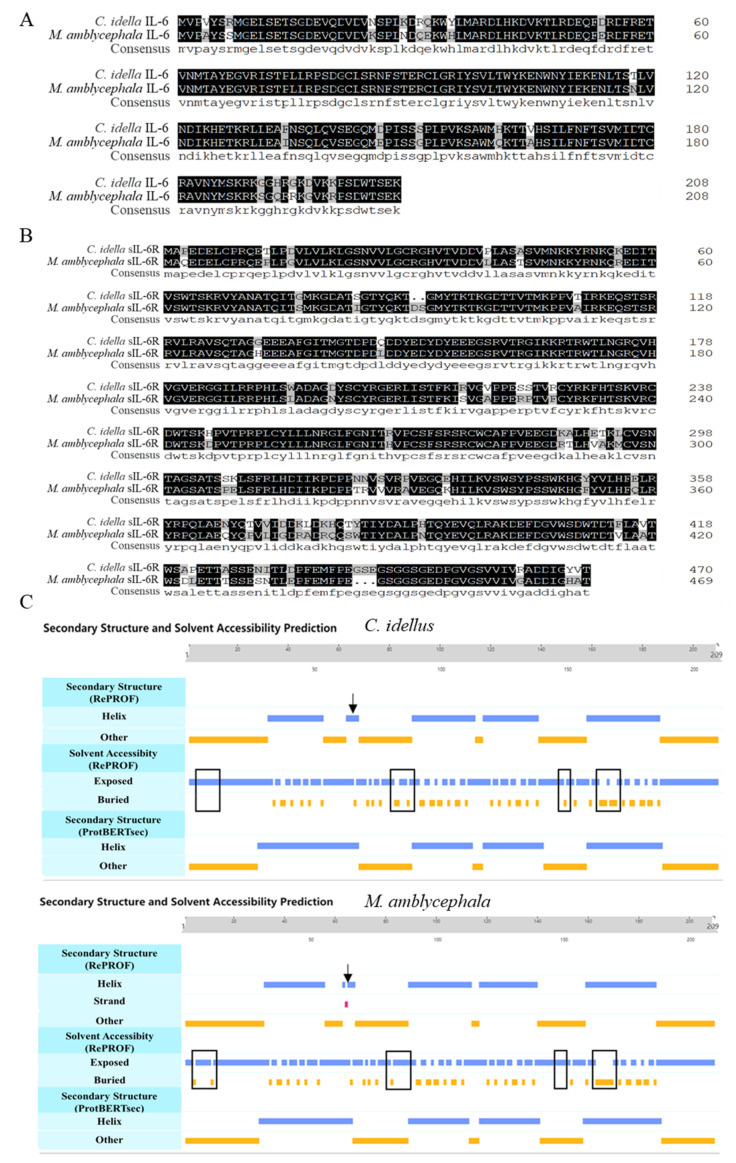
Sequence analysis of IL-6 and sIL-6R in grass carp (*C. idellus*) and blunt snout bream (*M. amblycephala*). Sequence alignment of ciIL-6 and maIL-6 (**A**), cisIL-6R and masIL-6R (**B**), and secondary structure and solvent accessibility prediction for ciIL-6 and maIL-6 (**C**). The consensus sequence amino acids are shown in solid black. The differences in solvent accessibility are boxed, and the difference in secondary structure is indicated by arrows.

To analyze homology of IL-6 between grass carp (*C. idellus*) and blunt snout bream (*M. amblycephala*), multiple sequence alignment was performed. The analysis showed that the similarity in amino acid sequence of IL-6 and sIL-6R between blunt snout bream and grass carp was as high as 90.91% and 86.89%, respectively (Figure 1A,B). However, the IL-6 proteins from grass carp and blunt snout bream showed some differences in secondary structure and solvent accessibility (Figure 1C).

### 2.2. Effects of Recombinant IL-6 on the Expression of Downstream Genes in L8824 Cells

**Figure 2:**
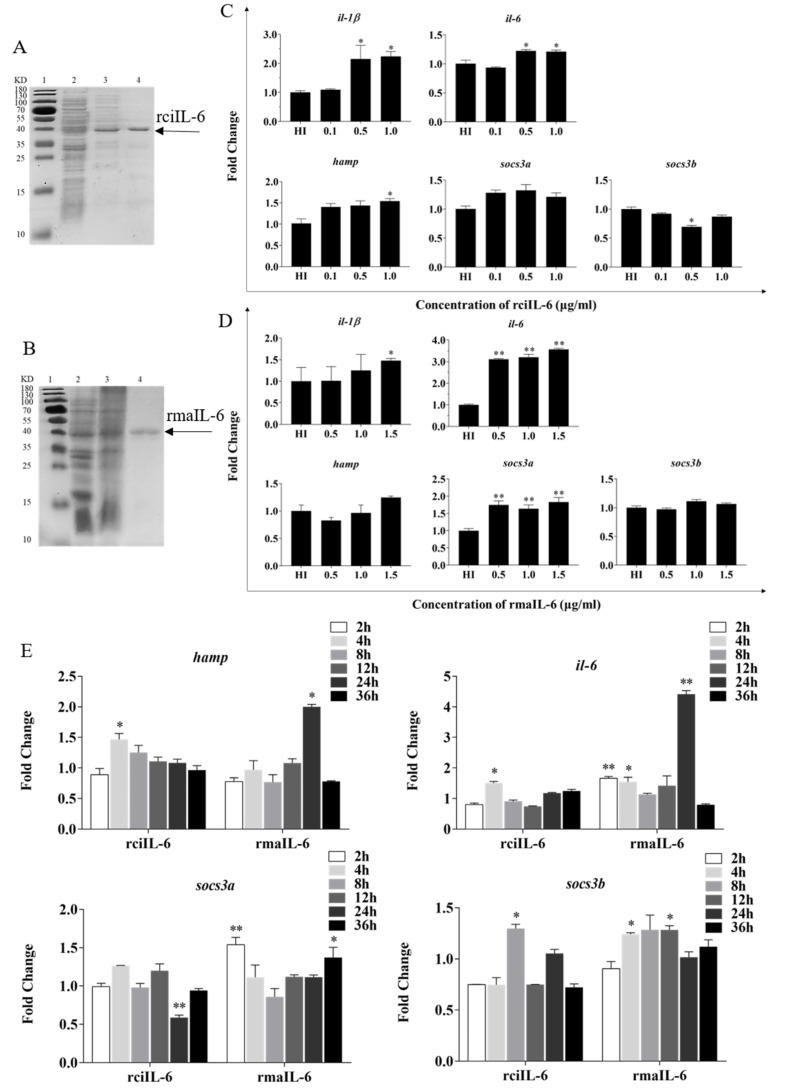
Production and biological activity of recombinant IL-6 of grass carp (rciIL-6) and blunt snout bream (rmaIL-6). SDS-PAGE of rciIL-6 (**A**) and rmaIL-6 (**B**) proteins. Lane 1: molecular mass marker; Lane 2: whole-cell lysate of non-induced *E. coli*; Lane 3: whole-cell lysate of induced *E. coli* containing the recombinant proteins; Lane 4: purified and refolded recombinant proteins. L8824 cells were treated with different concentrations of rciIL-6 (**C**) or rmaIL-6 (**D**). L8824 cells were treated with different concentrations of rciIL-6 or mail-6 for different times (**E**). The *hamp*, *il-6*, *il-1β*, *socs3a*, and *socs3b* mRNA were quantified by qPCR. Gene expression was normalized relative to the reference gene *18S rRNA*. Fold changes were calculated by comparing the average gene expression of the treatment groups with that of the corresponding control groups (HI, heat-inactivated protein). (**C**,**D**) Student’s *t*-test was used to determine the significance of differences between the experimental and the control groups. (**E**), One-way analysis of variance (ANOVA) was used to analyze the differences among different time points. Data are presented as mean ± SEM of at least three replicates for each experiment. * *p* < 0.05, ** *p* < 0.01.

As shown in Figure 2, recombinant grass carp IL-6 (rciIL-6) and recombinant blunt snout bream IL-6 (rmaIL-6) proteins were successfully produced in the inclusion bodies of *Escherichia coli*. The purified rciIL-6 (Figure 2A) and rmaIL-6 (Figure 2B) were visualized by similar single bands around 40 kDa (theoretical MW: 24.27 kDa for rciIL-6 or 24.15 kDa for rmaIL-6 and 18.3 kDa of pET-32a plasmid tag protein) on an SDS-PAGE gel.

To evaluate the biological activity of recombinant IL-6, the expression of downstream genes including *hamp*, *il-1β*, *il-6*, *socs3a*, and *socs3b* in L8824 cells was analyzed. As shown in Figure 2C, after 4 h of stimulation, the expression of *hamp il-1β* and *il-6* was induced by 0.5 and 1.0 μg/mL of rciIL-6 protein, while *socs3b* expression was inhibited by 0.5 μg/mL of rciIL-6. Similarly, as shown in Figure 2D, after 2 h of stimulation, the expression of *hamp* and *socs3b* could not be significantly modulated by rmaIL-6, but *il-6* (at all three doses), *il-1β* (at 1.5 μg/mL), and *socs3a* (at all three doses) could be induced by rmaIL-6.

As shown in Figure 2E, when L8824 cells were treated with rciIL-6 for different times, the mRNA level of *hamp* was significantly upregulated at 4 h and then gradually decreased to the control level, whereas, after rmaIL-6 treatment, the expression of *hamp* peaked at 24 h. In addition, rciIL-6 increased the *il-6* mRNA level only at 4 h, while rmaIL-6 could significantly upregulate the expression of *il-6* mRNA at 2, 4, and 24 h of stimulation. rciIL-6 significantly downregulated *socs3a* at 24 h, whereas rmaIL-6 significantly upregulated *socs3a* expression at 2 h and 36 h. In addition, rciIL-6 significantly increased *socs3b* expression only at 8 h, while rmaIL-6 significantly induced *socs3b* expression at both 4 h and 12 h.

### 2.3. Activation of Signaling Pathways by rmaIL-6 with or without RmasIL-6R

**Figure 3:**
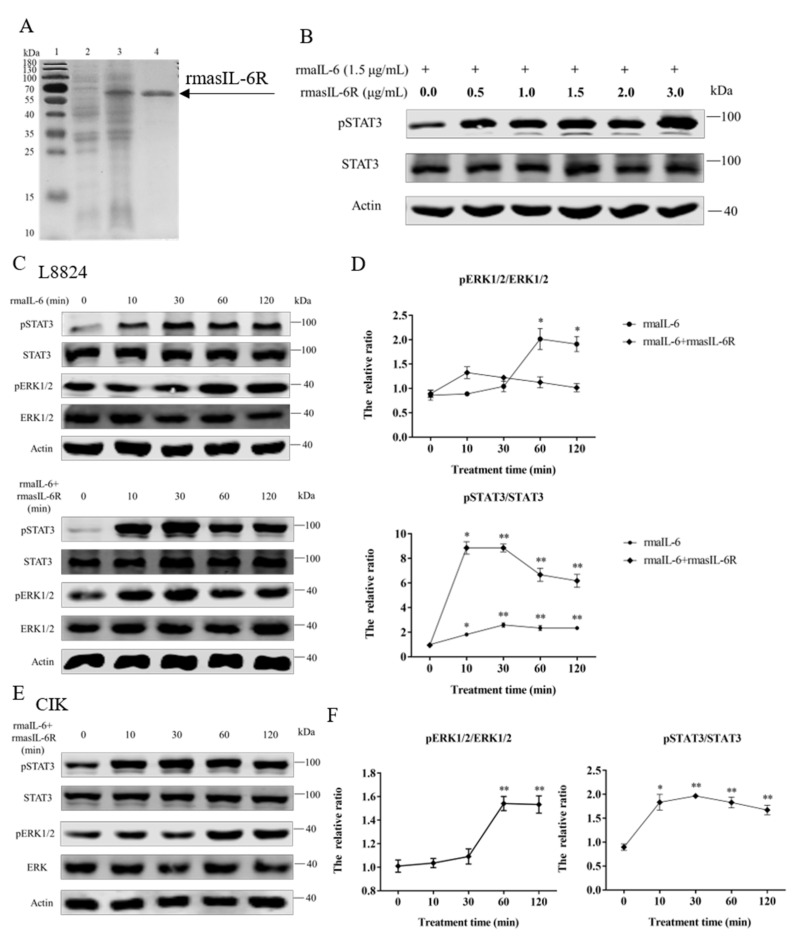
(**A**), SDS-PAGE of the rmasIL-6R produced from *E. coli*. Lane 1: molecular mass marker; Lane 2: whole-cell lysate of non-induced *E. coli*; Lane 3: whole-cell lysate of induced *E. coli* containing the recombinant protein; Lane 4: purified and refolded recombinant protein. (**B**), STAT3 phosphorylation in CIK cells treated with rmaIL-6+rmasIL-6R for 30 min. (**C**), Phosphorylation of STAT3 and ERK1/2 in L8824 cells treated with rmaIL-6 alone or in combination with rmasIL-6R. (**E**), Phosphorylation of STAT3 and ERK1/2 in CIK cells treated with rmaIL-6+rmasIL-6R. The signals of phosphorylated proteins and total proteins were first normalized to β-actin, and the ratios between phosphorylated protein and total protein were calculated. Data are presented as mean ± SEM of at least three replicates for each experiment (**D**,**F**). * *p* < 0.05, ** *p* < 0.01.

As shown in Figure 3A, rmasIL-6R was successfully obtained. The SDS-PAGE results revealed that the molecular mass of rmasIL-6R is about 60 kDa (the predicted MW of rmasIL-6R is 52.78 kDa, and that of His-tag is ~4.8 kDa), which is consistent with the theoretical molecular mass.

The *gp130* CDS fragment could be detected in L8824 cells, CIK cells, and primary hepatocytes cDNA template, and the *il-6r* CDS fragment could be detected in L8824 cells and primary hepatocytes cDNA template, but not in CIK cells (Supplementary Figure S2). As shown by western blot, in L8824 cells, STAT3 phosphorylation was induced by rmaIL-6 at μg/mL and 1.5 μg/mL, but rmaIL-6 at 1.5 μg/mL concentration was more effective. (Supplementary Figure S3). In CIK cells, STAT3 phosphorylation was induced by rmaIL-6 combined with lower concentrations of rmasIL-6R (i.e., 0.5 μg/mL) (Figure 3B). 

In order to investigate the involvement of rmaIL-6 in the JAK/STAT3 signaling pathway, L8824 cells were treated with rmaIL-6 alone (classical signaling) or together with rmasIL-6R (trans-signaling) for different times. The phosphorylation of ERK1/2 and STAT3 was then analyzed using western blot. The results showed that rmaIL-6 alone or rmaIL-6+rmasIL-6R could induce STAT3 phosphorylation. Although rmaIL-6+rmasIL-6R elicited stronger STAT3 phosphorylation than rmaIL-6 alone, both treatments presented similar kinetics, with phosphorylation peaking after 10–30 min of stimulation (Figure 3C). In addition, rmaIL-6 alone could induce ERK1/2 phosphorylation, peaking at 60 min after treatment. However, ERK1/2 phosphorylation did not change after treatment with rmaIL-6+rmasIL-6R (Figure 3D).

On the other hand, when CIK cells was stimulated with rmaIL-6 and rmasIL-6R alone or jointly, it was found that only rmaIL-6+rmasIL-6R could induce STAT3 phosphorylation (Supplementary Figure S3). Therefore, the phosphorylation of ERK1/2 and STAT3 was detected at different time points after CIK cells were stimulated only by rmaIL-6+rmasIL-6R (Figure 3E). As shown in Figure 3F, CIK cells responded to rmaIL-6 stimulation similarly to L8824 cells, where STAT3 phosphorylation increased significantly at 10 min, peaked at 30 min, and then declined slowly. We then investigated whether IL-6 induced activation of MEK/ERK signaling pathways by measuring the level of the ERK1/2 phosphorylation. Treatment with rmaIL-6+rmasIL-6R caused a strong phosphorylation of ERK1/2 in CIK cells at 60 and 120 min (Figure 3F).

**Figure 4:**
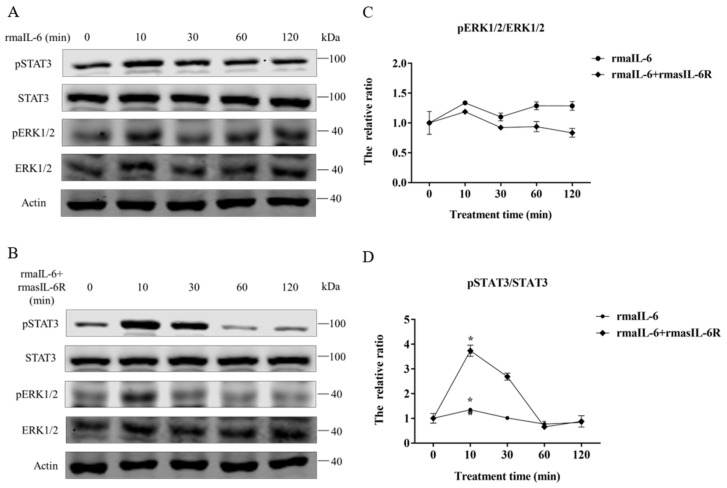
Phosphorylation of STAT3 and ERK1/2 in primary hepatocytes treated with rmaIL-6 alone (**A**) or in combination with rmasIL-6R (**B**). The signals of phosphorylated proteins and total proteins were first normalized to β-actin, and the ratios between phosphorylated protein and total protein were calculated (**C**,**D**). Data are presented as mean ± SEM of at least three replicates for each experiment. * *p* < 0.05.

In primary hepatocytes of grass carp, STAT3 phosphorylation was induced by rmaIL-6 or rmaIL-6+rmasIL-6R (Figure 4A,B). The stimulation of primary hepatocytes with rmaIL-6 or rmaIL-6+rmasIL-6R presented a similar kinetics, with a peak of STAT3 phosphorylation at 10 min (Figure 4C). However, neither rmaIL-6 nor rmaIL-6+rmasIL-6R could significantly affect ERK1/2 phosphorylation (Figure 4D).

**Figure 5:**
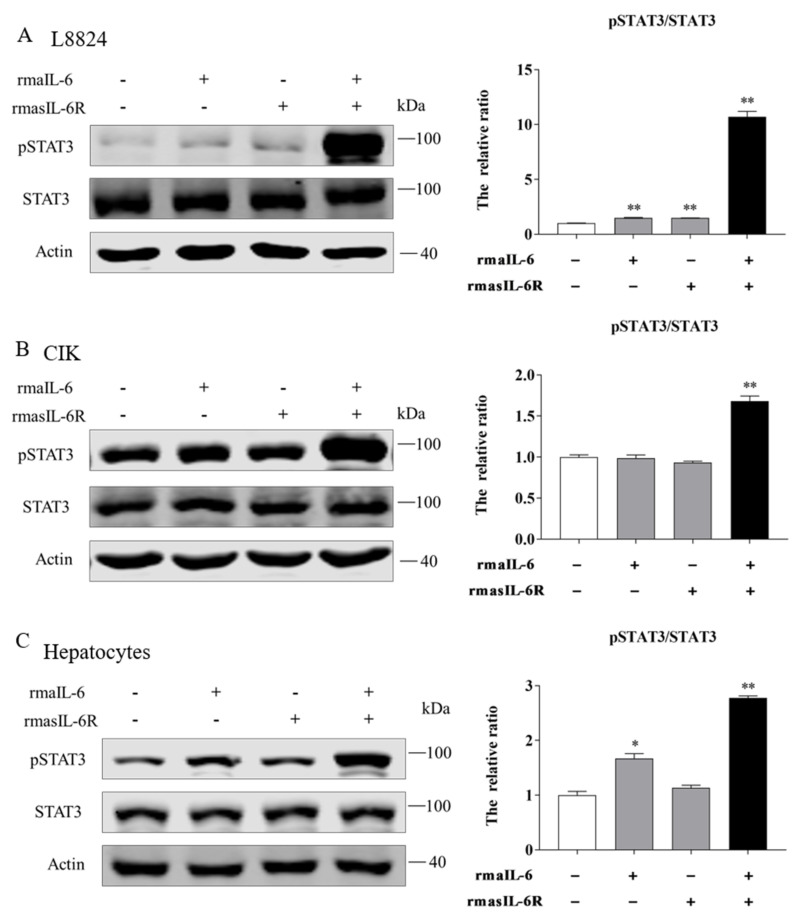
STAT3 phosphorylation in response to rmaIL-6 stimulation in CIK cells (**A**), L8824 cells (**B**), and primary hepatocytes (**C**). A representative blot containing phosphorylated proteins, total proteins, and β-actin is shown for each pathway (left column). Ratios of phosphorylated proteins to total proteins were calculated. Data are presented as mean ± SEM of at least three replicates for each experiment (right column). * *p* < 0.05, ** *p* < 0.01.

Moreover, in L8824 cells, STAT3 phosphorylation could be induced by rmaIL-6 alone or in combination with rmasIL-6R, but the effect of the combined stimulation was stronger (Figure 5A). In contrast, CIK cells responded differently to rmaIL-6 stimulation than L8824 cells. In CIK cells, STAT3 phosphorylation could not be induced by rmaIL-6 or rmasIL-6R alone, but only by their combination (Figure 5B). Similar to L8824 cells, STAT3 phosphorylation was induced in primary hepatocytes by rmaIL-6 alone or in combination with rmasIL-6R (Figure 5C)

### 2.4. RmaIL-6 Trans-Signaling Regulates Socs3a and Socs3b Expression via the JAK2/STAT3 Pathway in L8824 Cells and CIK Cells

**Figure 6:**
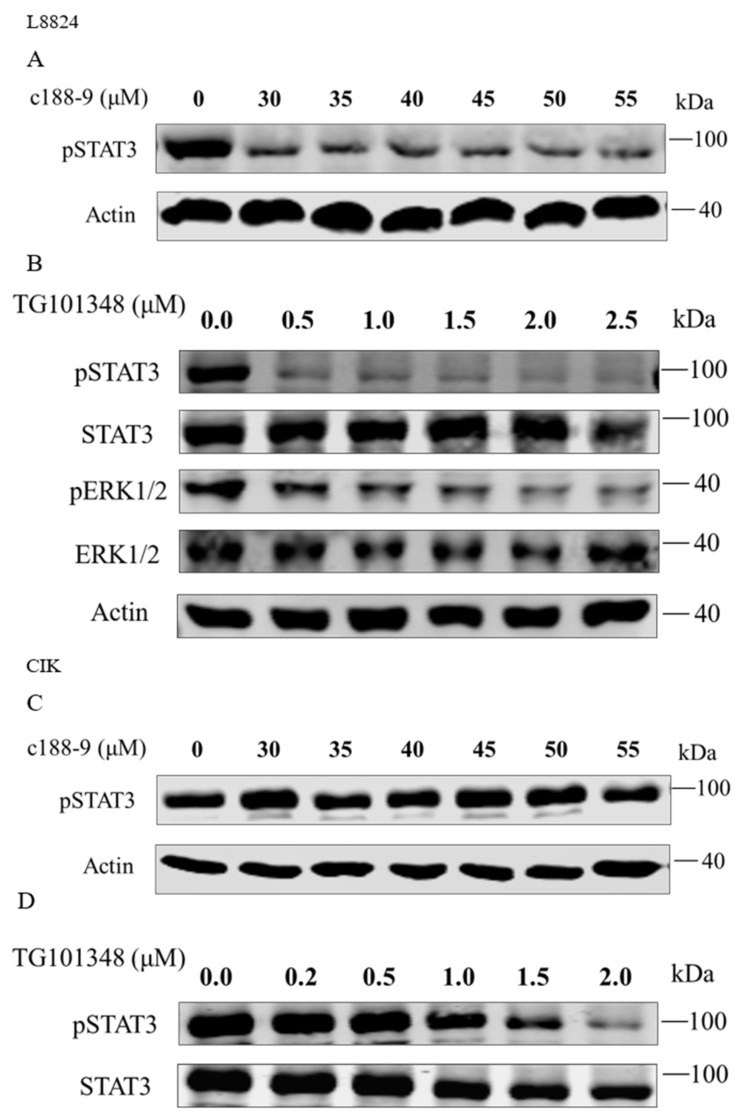
Inhibitory effects of c188-9 or TG101348 on the phosphorylation of STAT3 and ERK1/2. STAT3 phosphorylation after treatment with different concentrations of c188-9 in L8824 (**A**) and CIK cells (**C**). Phosphorylation of STAT3 and ERK1/2 after treatment with different concentrations of TG101348 in L8824 (**B**) and CIK (**D**) cells.

To further elucidate the regulation of downstream genes by rmaIL-6 trans-signaling, pharmacological inhibitors were used to interfere with the JAK2/STAT3 pathways, and the expression of *il-6*, *hamp*, *socs3a*, and *socs3b* was assessed after treatment by rmaIL-6+rmasIL-6R. First, the effects of inhibitors on the phosphorylation of STAT3 or ERK1/2 were examined. As expected, pretreatment of L8824 cells with the STAT3 inhibitor c188-9 inhibited STAT3 phosphorylation, and the JAK2 inhibitor TG101348 strongly inhibited the phosphorylation of STAT3 and ERK1/2 in a dose-dependent manner (Figure 6A,B). Both low and high concentrations of c188-9 did not significantly inhibit STAT3 phosphorylation in CIK cells, while TG101348 could inhibit STAT3 phosphorylation in a dose-dependent manner in CIK cells (Figure 6C,D).

**Figure 7:**
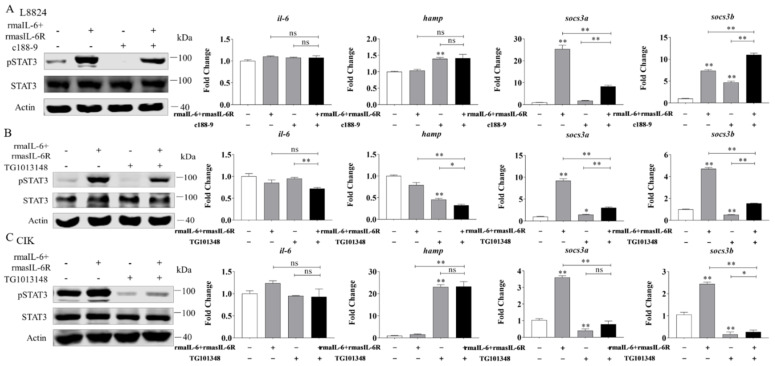
Effects of different inhibitors on IL-6 trans-signaling. (**A**), Effect of c188-9 on STAT3 phosphorylation (left) and gene expression (right) regulated by rmaIL-6+rmasIL-6R in L8824 cells. (**B**), Effect of TG101348 on STAT3 phosphorylation (left) and gene expression (right) in the downstream pathway activated by rmaIL-6+rmasIL-6R in L8824 cells. (**C**), Effect of TG101348 on STAT3 phosphorylation (left) and gene expression (right) regulated by rmaIL-6+rmasIL-6R in CIK cells. Data are presented as mean ± SEM of at least three replicates for each experiment. * *p* < 0.05, ** *p* < 0.01, ^ns^ no significance.

Next, L8824 cells were treated with rmaIL-6+rmasIL-6R after pretreatment with c188-9 or TG101348 for 10 h. As previously shown, in L8824 cells, rmaIL-6+rmasIL-6R could promote STAT3 phosphorylation, while c188-9 reduced STAT3 phosphorylation caused by rmaIL-6+rmasIL-6R (Figure 7A, left). In line with that, qPCR analysis showed that *socs3a* mRNA induced by rmaIL-6+rmasIL-6R was not detected after c188-9 treatment, while the expression of *socs3b* was further enhanced by c188-9. Besides, rmaIL-6+rmasIL-6R did not induce the expression of *il-6* and *hamp*, although c188-9 did induce the upregulation of *hamp* mRNA (Figure 7A, right). Similarly, treating L8824 cells with TG101348 prior to stimulation with rmaIL-6+rmasIL-6R resulted in the suppression of STAT3 phosphorylation (Figure 7B, left). Consistent with this, the induction of *socs3a* and *socs3b* by rmaIL-6+rmasIL-6R was inhibited by TG101348 pretreatment (Figure 7B, right). 

In CIK cells, pretreatment with the JAK2 inhibitor TG101348 blocked STAT3 phosphorylation induced by rmaIL-6+rmasIL-6R (Figure 7C, left). Inevitably, TG101348 pretreatment could inhibit the expression of *socs3a* and *socs3b* induced by rmaIL-6+rmasIL-6R. Surprisingly, *hamp* expression was strongly induced by TG101348 (Figure 7C, right). 

## 3. Discussion

Cytokines play an important role in the immune system. During IL-6 stimulation, STAT3 phosphorylation increased, while persistent activation of STAT3 contributed to IL-6 production in human basal cells [43]. In this study, rciIL-6 and rmaIL-6 could induce the expression of *il-6* in L8824 cells, similar to what observed in rainbow trout [37]. Therefore, IL-6 can increase *il-6* expression in an autocrine or paracrine fashion and may amplify and exacerbate the inflammatory response. In our work, both rciIL-6 and rmaIL-6 significantly upregulated the expression of *il-1β*. Previous studies in teleost showed that recombinant IL-6 protein could not affect the expression of *il-1β* in *L. crocea* after 24 h of stimulation [34] and even significantly reduced the expression of *il-1β* and *socs3* in rainbow trout at 24 h [37]. In stark contrast, IL-6 rapidly and dramatically induced *il-1β* expression in *Acipenser baeri* (Brandt, 1869) spleen 6 h after treatment [44]. These differences may be due to the different durations of IL-6 stimulation.

IL-6 has been shown to be a necessary and sufficient cytokine to induce *hamp* expression in mice, human hepatocytes, and cortical neurons [45,46]. Recombinant IL-6 induced the expression of *hamp* in rainbow trout macrophages [37]. Our results showed that rciIL-6 could rapidly induce the upregulation of *hamp* in L8824 cells. In addition, rmaIL-6 had no significant effect on *hamp* expression in a short time but could significantly upregulate *hamp* at 24 h in L8824 cells. In fish, *socs3* is associated with immune regulation as its expression is modulated by inflammatory stimulants, cytokines, and infection [47]. In our work, both rciIL-6 and rmaIL-6 significantly upregulated the expression of *socs3b*, but rciIL-6 inhibited the expression of *socs3a* at a certain time point. Therefore, IL-6 in teleost might play both pro-inflammatory and anti-inflammatory roles, but the mechanism is slightly diverse in different species. This difference may be due to structural differences or to a different refolding efficiency of the recombinant proteins.

The general opinion is that IL-6R is present in a few cell types, such as immune cells and hepatocytes, which are directly activated by IL-6 classical signaling [19,48]. In this study, we provide evidence of the existence of membrane-bound IL-6R in L8824 cells but not in CIK cells. IL-6R is important for ligand binding, but it has only a short cytoplasmic domain, and its signal transduction is dependent on the recruitment of gp130 [10,49]. IL-6 is generally believed to activate the JAK/STAT3 pathway, through either soluble or membrane-bound IL-6R. Consistent with this, we found that both rmaIL-6 classical signaling and trans-signaling could trigger STAT3 phosphorylation in a time-dependent manner. However, trans-signaling led to more intense STAT3 phosphorylation than classical signaling. This is also consistent with relevant research in mammals [50,51]. In addition, studies have shown that IL-6-mediated downstream signaling cascade pathways mainly include the JAK/STAT3, MEK/ERK, and PI3K/AKT pathways [52,53,54]. Here, we reported the difference between two signals mediated by rmaIL-6 in different cells. In L8824 cells, classical signaling involves both JAK/STAT3 and MEK/ERK pathways, whereas trans-signaling involves only the JAK/STAT3 pathway. In contrast, in CIK cells, IL-6 trans-signaling could activate both JAK/STAT3 and MEK/ERK pathways. In mammals, several pieces of evidence indicated reciprocal crosstalk between the MEK/ERK pathway and the JAK/STAT3 pathway [55,56]. In addition, IL-6-type cytokines did not activate ERK1/2, but activated STAT3 in some human cells [57,58]. In primary hepatocytes, both IL-6 classical signaling and trans-signaling could activate the JAK/STAT3 pathway but not the MEK/ERK pathway. These results suggest that IL-6 is critical to the activation of the JAK/STAT3 pathway and may not be key to the activation of the MEK/ERK pathway in grass carp cells. Meanwhile, a strong activation of STAT3 may affect ERK phosphorylation to prevent over-immunity in teleost, which is beneficial to maintain the normal operation of the immune system.

It is well known that activation of the JAK/STAT3 pathway leads to STAT3 dimerization and translocation into the nucleus, where it initiates gene transcription [59]. It was shown that *socs3* transcription induced by IL-6 lasted at least 48 h in HUVECs cells [60]. In L8824 cells, STAT3 was found to be essential for trans-signaling-mediated expression of *socs3a* and *socs3b*. Besides, in L8824 cells and CIK cells, blockade of JAK2 also resulted in complete inhibition of STAT3 phosphorylation as well as of *socs3a* and *socs3b* expression induced by trans-signaling. These findings indicate that JAK2 is located upstream of STAT3 in the signaling pathway mediated by IL-6 trans-signaling and that JAK2 is crucial for the induction of *socs3a* and *socs3b*. The JAK2/STAT3 inhibitor AG490 reduced *hamp* mRNA expression even when the cells were exposed to IL-6 [61]. In our study, the expression of *hamp* was not affected by the rmaIL-6 trans-signaling pathway in the short time, but TG101348 could significantly change its expression in L8824 cells and CIK cells. In previous studies, the JAK inhibitor also acted on other signaling pathways such as MEK/ERK and PI3K/AKT [62,63]. However, whether TG101348 affects *hamp* expression by inhibiting other signal pathways needs further study.

## 4. Materials and Methods

### 4.1. Cell Lines and Fish

Because IL-6 and sIL-6R proteins are conserved between grass carp and blunt snout bream, and blunt snout bream has no stable cell line, grass carp hepatic (L8824) cells and grass carp kidney (CIK) cells (Cell Collection Centre for Freshwater Organisms of Huazhong Agricultural University, Wuhan, China) were selected as model cells in this study. L8824 cells and CIK cells were cultured in M199 medium containing 10% fetal bovine serum with 100 U/mL penicillin and streptomycin (Gibco, NY, USA) and were kept at 28 °C in a 5% CO_2_ environment.

To amplify the blunt snout bream *il-6* (*mail-6*), *il-6r* (*mail-6r*) and grass carp *il-6* (*ciil-6*) cDNA sequences, liver samples from grass carp and blunt snout bream were obtained. Healthy blunt snout bream (0.5–0.7 kg) and grass carp (1.0–1.5 kg) used in the study were obtained from Fisheries College Aquaculture Base, Huazhong Agricultural University, China.

### 4.2. Isolation and Culture of Hepatocytes

In this study, primary hepatocytes of grass carp were isolated and cultured according to a previous study [64]. Briefly, prior to the isolation of hepatocytes, the blood of the fish was drawn with a syringe. Then, the liver was rapidly isolated and washed several times in ice-cold phosphate-buffered saline (PBS) (Servicebio, Wuhan, China) containing 500 U/mL penicillin and streptomycin. After removal of PBS using sterile pipettes, the samples were cut into small pieces (about 1 mm^3^). The small pieces of liver were digested with trypsin at 28 °C for 10 min, then the cells were collected, and the process was repeated 3 times. Thereafter, the cell suspension was centrifuged at 400 g for 10 min and washed twice. The harvested cell pellets were resuspended in M199 medium (Gibco, NY, USA) with 10% fetal bovine serum (Gibco, NY, USA) and 100 U/mL penicillin and streptomycin (Gibco, NY, USA) at a density of 1 × 10^6^ cells/mL. Finally, primary hepatocytes were kept at 28 °C in a 5% CO_2_ environment.

### 4.3. Sequence Obtainment and Analysis

The mRNA sequences of *il-6* (GenBank: KC535507.1), *hamp* (GenBank: JQ246442.1), *socs3a* (GenBank: MT925699.1), *socs3b* (GenBank: MH463452.1) of grass carp and *il-6* (GenBank: KJ755058.1), *il-6r* (GenBank: RXFO00000000.1) of blunt snout bream were derived from NCBI (https://www.ncbi.nlm.nih.gov/nuccore/, accessed on 15 March 2021). The amino acid sequences of IL-6 (GenBank: AHB51767.1), IL-6R (GenBank: AVI05199.1) of grass carp and IL-6 (GenBank: AIG51270.1) of blunt snout bream were derived from NCBI. The blunt snout bream IL-6R CDS and amino acid sequence were deduced using the Open Reading Frame Finder on the NCBI website (http://www.ncbi.nlm.nih.gov/gorf/orfig.cgi, accessed on 10 August 2020). The protein domain features were predicted by using the Simple Modular Architecture Research Tool (SMART) (http://smart.embl-heidelberg.de/, accessed on 9 August 2021). BLASTN and BLASTP (http://www.ncbi.nlm.Nih.gov/BLAST/) (25 June 2021) were used to identify homologous sequences. Multiple amino acid sequences were aligned using the DNAMAN V6 software (LynnonBiosoft, San Ramon, CA, USA). The protein secondary structure and solvent accessibility were predicted by predictprotein (https://predictprotein.org/home, accessed on 9 August 2021).

### 4.4. RNA Extraction and cDNA Synthesis

Total RNA was extracted with RNAiso Plus (Takara, Shiga, Japan) according to the manufacturer’s instructions. The concentration and quality of total RNA were estimated by means of spectrophotometry with NanoDrop 2000 (Thermo Scientific, Delaware, Waltham, MA, USA) and agarose gel electrophoresis. For quantitative PCR (qPCR) analysis, 1 μg of total RNA was reverse-transcribed using the PrimeScript^®^ RT reagent Kit (Takara, Shiga, Japan) and then stored at −20 °C for further use.

### 4.5. Expression and Purification of the Recombinant Proteins ciIL-6, maIL-6, and masIL-6R

The mature peptide-coding sequences of *ciil-6*, *mail-6*, and *masil-6r* were amplified by reverse-transcriptase polymerase chain reaction (RT-PCR) using the liver cDNA of grass carp or blunt snout bream as a template. The specific gene primers are listed in Supplementary Table S1. The amplified products were digested by *Eco*R I/*Xho* I, *Bam*H I/*Hin*d III, and *Eco*R I/*Hin*d III, respectively, then ligated into pET-28a/pET-32a, and transfected into BL21 cells (DE3; Tsingke, Jiangsu, China).

The colonies were inoculated into 500 mL of Luria–Bertani (LB) medium containing ampicillin (Amp) or kanamycin (Kan) (50 μg/mL), and the culture solution was incubated at 200 r/min and 37 °C until the OD600 value was 0.5–0.6. Then, the recombinant proteins were induced with isopropyl-β-D-thiogalactoside (IPTG) for 10–12 h prior to harvest. After ultrasonication, the recombinant proteins were affinity-purified using the His-Tagged Inclusion Body Protein Purification Kit (CoWin Biosciences, China) according to the manufacturer’s instructions. The proteins were analyzed by SDS-PAGE and visualized after staining with Coomassie brilliant blue R-250. Then, the purified recombinant proteins were dialyzed and refolded. The concentrations of the recombinant proteins were determined using NanoDrop 2000 (Thermo Scientific, Delaware, Waltham, MA, USA). The recombinant proteins were aliquoted and stored at −80 °C for further use.

### 4.6. Treatment of Cells

L8824 cells (2.5 × 10^5^ cells/well for 6-well plates) or CIK cells (4.0 × 10^5^ cells/well for 6-well plates) were cultured overnight before treatment. To evaluate the biological activity of recombinant IL-6, L8824 cells were treated with different concentrations of rciIL-6 (0.0, 0.1, 0.5, and 1.0 μg/mL) for 4 h or with rmaIL-6 (0.0, 0.5, 1.0, and 1.5 μg/mL) for 2 h. Then, 1.0 μg/mL rciIL-6 and 1.5 μg/mL rmaIL-6 were selected for the follow-up experiments. Whereafter, L8824 cells and primary hepatocytes were incubated with 1.0 μg/mL rciIL-6 and 1.5 μg/mL rmaIL-6 for different times (2, 4, 8, 12, 24, and 36 h), respectively. 

To evaluate the biological activity of rmasIL-6R, CIK cells were treated with rmaIL-6+rmasIL-6R (0.0, 0.5, 1.0, 1.5, 2.0, and 3.0 μg/mL) for 30 min. Then, 1.0 μg/mL rmasIL-6R was selected for the follow-up experiments. After that, L8824 cells, CIK cells, and primary hepatocytes were incubated with rmaIL-6+rmasIL-6R for different times (0, 10, 30, 60, and 120 min). Moreover, L8824 cells and CIK cells were treated with rmaIL-6, rmasIL-6R, and rmaIL-6+rmasIL-6R for 30 min. 

The STAT3 inhibitor c188-9 and the JAK2 inhibitor TG101348 were purchased from Selleck (China). Different concentrations of c188-9 (0, 30, 35, 40, 45, 50, and 55 μM) or TG101348 (0.0, 0.2, 0.5, 1.0, 1.5, 2.0, and 2.5 μM) were added into the culture medium, and the cultures were incubated for 10 h to select the proper concentrations for the subsequent treatments. Besides, the cells were retreated with or without rmaIL-6+rmasIL-6R after pretreatment with 35 μM c188-9 and 0.5 μM (L8824 cells) or 2.0 μM (CIK cells) TG101348 for 10 h. 

All the above experiments were set up with a blank control and three repetitions. After treatments, the cells were collected to extract total RNA or protein.

### 4.7. qPCR Analysis

qPCR was performed in a Bio-Rad CFX Connect™ real-time PCR system (Bio-Rad, US). The qPCR mixture consisted of 1.0 μL cDNA template, 7.4 μL nuclease-free water, 10.0 μL LightCycler^®^ 480 SYBR Green I Master (Roche, Switzerland), and 0.8 μL of each forward and reverse primers (10 μM). qPCR was conducted using the following program: 95 °C for 5 min, 40 cycles of 95 °C for 5 s, 60 °C for 20 s, and 72 °C for 20 s, followed by melting curve determination from 65 °C to 95 °C to verify the amplification of a single product. The relative expression levels of the target genes were measured by the 2^−ΔΔCt^ method [65], and *18S rRNA* was used as the internal control [66,67,68,69]. The relative expression levels were indicated as fold change. Plasmid construction (ciIL-6, maIL-6, and masIL-6R) and qPCR primers (*il-1β*, *il-6*, *hamp*, *socs3a*, *socs3b*, and *18S rRNA*) are shown in Supplementary Table S1.

### 4.8. Protein Extraction and Quantification

The cells were rinsed with PBS and lysed using RIPA lysis buffer (Beyotime, Shanghai, China). To quantify the proteins, the BCA Protein Assay kit was used (Beyotime, Shanghai, China) according to the manufacturer’s instructions, and absorbance at 540 nm was measured using Multiskan-Ascent (Tecan NanoQuant 200, Tecan, Switzerland).

### 4.9. Western Blot

Cell lysates were mixed with 5 × SDS sample buffer and denatured for 10 min at 95 °C. Next, the protein mixture was loaded into an 8% SDS–PAGE gel, then transferred to the NC membranes (Pall, St. Show Low, AZ, USA) at 200 mA for 1 h. Subsequently, the membranes were blocked with TBST buffer containing 5% BSA or skimmed milk powder for 1.5 h at room temperature, then incubated with anti-STAT3, anti-ERK1/2 (Proteintech, Rosemont, IL, USA), anti-pSTAT3 (Huabio, Hangzhou, China), anti-pERK1/2, anti-β-actin (ABclonal, Wuhan, China) antibodies overnight at 4 °C. On the second day, the membranes were washed with TBST, incubated with goat anti-rabbit secondary antibodies (Yeasen, Shanghai, China) for 1 h at room temperature, and photographed using the Odyssey CLx image system (Li-cor, Lincoln, NE, USA). Finally, the gray value intensities of western blot results were measured by ImageJ software.

### 4.10. Statistical Analysis

Data are presented as mean ± standard error of the mean (SEM) of three repeated experiments. Statistical significance was analyzed using Student’s *t*-test or one-way analysis of variance (ANOVA); *p* < 0.05 indicated significant difference, and *p* < 0.01 was considered as indicating extremely significant difference.

## 5. Conclusions

To sum up, rmaIL-6 and rmasIL-6R have biological activity and activate the JAK/STAT3 pathway and the expression of downstream genes. In L8824 cells, IL-6 classical signaling activated both JAK/STAT3 and MEK/ERK pathways, whereas trans-signaling activated only the JAK/STAT3 pathway. In CIK cells, IL-6 trans-signaling activated both JAK/STAT3 and MEK/ERK pathways. In primary hepatocytes, IL-6 classical signaling and trans-signaling only activated the JAK/STAT3 pathway. Therefore, IL-6 mainly acts by activating the JAK/STAT3 pathway. In addition, we demonstrated that activation of the JAK2/STAT3 pathways is essential for IL-6 trans-signaling-induced *socs3a* and *socs3b* production in L8824 cells and CIK cells. This study adds to the understanding of the regulation mechanisms of IL-6 classical and trans-signaling in fish, enriches our knowledge of fish immunology, and provides a theoretical basis for the prevention and treatment of fish diseases in the future.

## Data Availability

All datasets generated for this study are included in the article/Supplementary Materials.

## Supplementary Materials

The following supporting information can be downloaded at: https://www.mdpi.com/article/10.3390/ijms23042019/s1.

## Abbreviations

IL-6	Interleukin-6
IL-6R	Interleukin-6 receptor
IL-1β	Interleukin-1β
Hamp	Hepcidin
SOCS3	Suppressor of cytokine signaling 3
ciIL-6	Grass carp IL-6
ciIL-6R	Grass carp IL-6R
maIL-6	Blunt snout bream IL-6
maIL-6R	Blunt snout bream IL-6R
masIL-6R	Blunt snout bream soluble IL-6R receptor
rciIL-6	Recombinant grass carp IL-6 protein
rmaIL-6	Recombinant blunt snout bream IL-6 protein
rmasIL-6R	Recombinant blunt snout bream soluble IL-6 receptor protein
JAK	Janus kinases
STAT	Signal transducer and activator of transcription
gp130	Glycoprotein 130
ERK	Extracellular-signal-regulated kinase
MEK	Mitogen-activated protein kinase
PI3K	Phosphatidylinositol 3 kinase
AKT	Protein kinase b
mIL-6R	Membrane-bound IL-6R

## References

[1] Hirano T., Yasukawa K., Harada H., Taga T., Watanabe Y., Matsuda T., Kashiwamura S., Nakajima K., Koyama K., Iwamatsu A. (1986). Complementary DNA for a novel human interleukin (BSF-2) that induces B lymphocytes to produce immunoglobulin. Nature.

[2] Ataie-Kachoie P., Pourgholami M.H., Richardson D.R., Morris D.L. (2014). Gene of the month: Interleukin 6 (IL-6). J. Clin. Pathol..

[3] Kimura A., Kishimoto T. (2010). IL-6: Regulator of Treg/Th17 balance. Eur. J. Immunol..

[4] Schmidt-Arras D., Rose-John S. (2016). IL-6 pathway in the liver: From physiopathology to therapy. J. Hepatol..

[5] Diehl S., Rincon M. (2002). The two faces of IL-6 on Th1/Th2 differentiation. Mol. Immunol..

[6] Tanaka T., Narazaki M., Kishimoto T. (2018). Interleukin (IL-6) Immunotherapy. Cold Spring Harb. Perspect. Biol..

[7] Ridgley L.A., Anderson A.E., Maney N.J., Naamane N., Skelton A.J., Lawson C.A., Emery P., Isaacs J.D., Carmody R.J., Pratt A.G. (2019). IL-6 mediated transcriptional programming of naive CD4+ T Cells in early rheumatoid arthritis drives dysregulated effector function. Front. Immunol..

[8] Tanaka T., Narazaki M., Kishimoto T. (2014). IL-6 in inflammation, immunity, and disease. Cold Spring Harb. Perspect. Biol..

[9] Akira S., Taga T., Kishimoto T. (1993). Interleukin-6 in biology and medicine. Adv. Immunol..

[10] Mihara M., Hashizume M., Yoshida H., Suzuki M., Shiina M. (2012). IL-6/IL-6 receptor system and its role in physiological and pathological conditions. Clin. Sci..

[11] Theurl I., Schroll A., Sonnweber T., Nairz M., Theurl M., Willenbacher W., Eller K., Wolf D., Seifert M., Sun C.C. (2011). Pharmacologic inhibition of hepcidin expression reverses anemia of chronic inflammation in rats. Blood.

[12] Yu H., Pardoll D., Jove R. (2009). STATs in cancer inflammation and immunity: A leading role for STAT3. Nat. Rev. Cancer.

[13] Long M.H., Zhang C., Xu D.Q., Fu W.L., Gan X.D., Li F., Wang Q., Xia W., Xu D.G. (2020). PM_2.5_ aggravates diabetes via the systemically activated IL-6-mediated STAT3/SOCS3 pathway in rats’ liver. Environ. Pollut..

[14] Yoon S., Woo S.U., Kang J.H., Kim K., Kwon M.H., Park S., Shin H.J., Gwak H.S., Chwae Y.J. (2010). STAT3 transcriptional factor activated by reactive oxygen species induces IL6 in starvation-induced autophagy of cancer cells. Autophagy.

[15] Marotta L.L., Almendro V., Marusyk A., Shipitsin M., Schemme J., Walker S.R., Bloushtain-Qimron N., Kim J.J., Choudhury S.A., Maruyama R. (2011). The JAK2/STAT3 signaling pathway is required for growth of CD44^+^CD24^−^ stem cell-like breast cancer cells in human tumors. J. Clin. Investig..

[16] Culig Z. (2013). Interleukin-6 as a therapy target in oral squamous carcinoma. Expert Opin Ther. Targets.

[17] Zegeye M.M., Lindkvist M., Falker K., Kumawat A.K., Paramel G., Grenegard M., Sirsjo A., Ljungberg L.U. (2018). Activation of the JAK/STAT3 and PI3K/AKT pathways are crucial for IL-6 trans-signaling-mediated pro-inflammatory response in human vascular endothelial cells. Cell Commun. Signal.

[18] Rose-John S., Waetzig G.H., Scheller J., Grotzinger J., Seegert D. (2007). The IL-6/sIL-6R complex as a novel target for therapeutic approaches. Expert Opin. Ther. Targets.

[19] Baran P., Hansen S., Waetzig G.H., Akbarzadeh M., Lamertz L., Huber H.J., Ahmadian M.R., Moll J.M., Scheller J. (2018). The balance of interleukin (IL)-6, IL-6·soluble IL-6 receptor (sIL-6R), and IL-6·sIL-6R·sgp130 complexes allows simultaneous classic and trans-signaling. J. Biol. Chem..

[20] Wolf J., Rose-John S., Garbers C. (2014). Interleukin-6 and its receptors: A highly regulated and dynamic system. Cytokine.

[21] Schumacher N., Meyer D., Mauermann A., von der Heyde J., Wolf J., Schwarz J., Knittler K., Murphy G., Michalek M., Garbers C. (2015). Shedding of endogenous interleukin-6 receptor (IL-6R) is governed by a disintegrin and metalloproteinase (ADAM) proteases while a full-length IL-6R isoform localizes to circulating microvesicles. J. Biol. Chem..

[22] Garbers C., Aparicio-Siegmund S., Rose-John S. (2015). The IL-6/gp130/STAT3 signaling axis: Recent advances towards specific inhibition. Curr. Opin. Immunol..

[23] Jones S.A., Rose-John S. (2002). The role of soluble receptors in cytokine biology: The agonistic properties of the sIL-6R/IL-6 complex. Biochim. Biophys. Acta.

[24] Rose-John S. (2012). IL-6 trans-signaling via the soluble IL-6 receptor: Importance for the pro-inflammatory activities of IL-6. Int. J. Biol. Sci..

[25] Schaper F., Rose-John S. (2015). Interleukin-6: Biology, signaling and strategies of blockade. Cytokine Growth Factor Rev..

[26] Redell M.S., Ruiz M.J., Alonzo T.A., Gerbing R.B., Tweardy D.J. (2011). Stat3 signaling in acute myeloid leukemia: Ligand-dependent and -independent activation and induction of apoptosis by a novel small-molecule Stat3 inhibitor. Blood.

[27] Li M., Gao J., Li D., Yin Y. (2018). CEP55 promotes cell motility via JAK2–STAT3–MMPs cascade in hepatocellular carcinoma. Cells.

[28] Li X., Mak V.C.Y., Zhou Y., Wang C., Wong E.S.Y., Sharma R., Lu Y., Cheung A.N.Y., Mills G.B., Cheung L.W.T. (2019). Deregulated Gab2 phosphorylation mediates aberrant AKT and STAT3 signaling upon PIK3R1 loss in ovarian cancer. Nat. Commun..

[29] Ogasawara K., Kam J., Thomas M., Liu L., Liu M., Xue Y., Surapaneni S., Carayannopoulos L.N., Zhou S., Palmisano M. (2021). Effects of strong and moderate CYP3A4 inducers on the pharmacokinetics of fedratinib in healthy adult participants. Cancer Chemother. Pharm..

[30] Varela M., Dios S., Novoa B., Figueras A. (2012). Characterisation, expression and ontogeny of interleukin-6 and its receptors in zebrafish (*Danio rerio*). Dev. Comp. Immunol..

[31] Bird S., Zou J., Savan R., Kono T., Sakai M., Woo J., Secombes C. (2005). Characterisation and expression analysis of an interleukin 6 homologue in the Japanese pufferfish, *Fugu rubripes*. Dev. Comp. Immunol..

[32] Nam B.H., Byon J.Y., Kim Y.O., Park E.M., Cho Y.C., Cheong J. (2007). Molecular cloning and characterisation of the flounder (*Paralichthys olivaceus*) interleukin-6 gene. Fish Shellfish Immunol..

[33] Castellana B., Iliev D.B., Sepulcre M.P., MacKenzie S., Goetz F.W., Mulero V., Planas J.V. (2008). Molecular characterization of interleukin-6 in the gilthead seabream (*Sparus aurata*). Mol. Immunol..

[34] Zhu Q., Li C., Yu Z.X., Zou P.F., Meng Q.X., Yao C.L. (2016). Molecular and immune response characterizations of IL-6 in large yellow croaker (*Larimichthys crocea*). Fish Shellfish Immunol..

[35] Iliev D.B., Castellana B., Mackenzie S., Planas J.V., Goetz F.W. (2007). Cloning and expression analysis of an IL-6 homolog in rainbow trout (*Oncorhynchus mykiss*). Mol. Immunol..

[36] Fu X., Ding Z., Fan J., Wang H., Zhou F., Cui L., Boxiang C., Wang W., Liu H. (2016). Characterization, promoter analysis and expression of the interleukin-6 gene in blunt snout bream, *Megalobrama amblycephala*. Fish Physiol. Biochem..

[37] Costa M.M., Maehr T., Diaz-Rosales P., Secombes C.J., Wang T. (2011). Bioactivity studies of rainbow trout (*Oncorhynchus mykiss*) interleukin-6: Effects on macrophage growth and antimicrobial peptide gene expression. Mol. Immunol..

[38] Chen H.H., Lin H.T., Foung Y.F., Han-You Lin J. (2012). The bioactivity of teleost IL-6: IL-6 protein in orange-spotted grouper (*Epinephelus coioides*) induces Th2 cell differentiation pathway and antibody production. Dev. Comp. Immunol..

[39] Kaneda M., Odaka T., Suetake H., Tahara D., Miyadai T. (2012). Teleost IL-6 promotes antibody production through STAT3 signaling via IL-6R and gp130. Dev. Comp. Immunol..

[40] Wang X., Guo Y., Wen C., Lv M., Gan N., Zhou H., Zhang A., Yang K. (2019). Molecular characterization of grass carp interleukin-6 receptor and the agonistic activity of its soluble form in head kidney leucocytes. Fish Shellfish Immunol..

[41] Zhou E., Yan F., Li B., Chen M., Tu X., Wu S., Wu H., Wei X., Fu S., Wu L. (2020). Molecular and functional characterization of IL-6 receptor (IL-6R) and glycoprotein 130 (gp130) in Nile tilapia (*Oreochromis niloticus*). Dev. Comp. Immunol..

[42] Zhang C.N., Zhang J.L., Liu W.B., Wu Q.J., Gao X.C., Ren H.T. (2016). Cloning, characterization and mRNA expression of interleukin-6 in blunt snout bream (*Megalobrama amblycephala*). Fish Shellfish Immunol..

[43] Chang R., Song L., Xu Y., Wu Y., Dai C., Wang X., Sun X., Hou Y., Li W., Zhan X. (2018). Loss of Wwox drives metastasis in triple-negative breast cancer by JAK2/STAT3 axis. Nat. Commun..

[44] Wang X., Chen J., Zhang R., Liu L., Ma G., Zhu H. (2020). Interleukin-6 in siberian sturgeon (*Acipenser baeri*): Molecular characterization and immune functional activity. Fish Shellfish Immunol..

[45] Nemeth E., Rivera S., Gabayan V., Keller C., Taudorf S., Pedersen B.K., Ganz T. (2004). IL-6 mediates hypoferremia of inflammation by inducing the synthesis of the iron regulatory hormone hepcidin. J. Clin. Investig..

[46] Qian Z.M., He X., Liang T., Wu K.C., Yan Y.C., Lu L.N., Yang G., Luo Q.Q., Yung W.H., Ke Y. (2014). Lipopolysaccharides upregulate hepcidin in neuron via microglia and the IL-6/STAT3 signaling pathway. Mol. Neurobiol..

[47] Wang T., Gao Q., Nie P., Secombes C.J. (2010). Identification of suppressor of cytokine signalling (SOCS) 6, 7, 9 and CISH in rainbow trout *Oncorhynchus mykiss* and analysis of their expression in relation to other known trout SOCS. Fish Shellfish Immunol..

[48] Modares N.F., Polz R., Haghighi F., Lamertz L., Behnke K., Zhuang Y., Kordes C., Haussinger D., Sorg U.R., Pfeffer K. (2019). IL-6 trans-signaling controls liver regeneration after partial hepatectomy. Hepatology.

[49] Heinrich P.C., Behrmann I., Muller-Newen G., Schaper F., Graeve L. (1998). Interleukin-6-type cytokine signalling through the gp130/Jak/STAT pathway. Biochem. J..

[50] Robinson M.B., Deshpande D.A., Chou J., Cui W., Smith S., Langefeld C., Hastie A.T., Bleecker E.R., Hawkins G.A. (2015). IL-6 trans-signaling increases expression of airways disease genes in airway smooth muscle. Am. J. Physiol. Lung Cell Mol. Physiol..

[51] Inoue-Mochita M., Inoue T., Kojima S., Futakuchi A., Fujimoto T., Sato-Ohira S., Tsutsumi U., Tanihara H. (2018). Interleukin-6-mediated trans-signaling inhibits transforming growth factor-beta signaling in trabecular meshwork cells. J. Biol. Chem..

[52] Klein C., Wustefeld T., Assmus U., Roskams T., Rose-John S., Muller M., Manns M.P., Ernst M., Trautwein C. (2005). The IL-6-gp130-STAT3 pathway in hepatocytes triggers liver protection in T cell-mediated liver injury. J. Clin. Investig..

[53] Pop V.V., Seicean A., Lupan I., Samasca G., Burz C.C. (2017). IL-6 roles—Molecular pathway and clinical implication in pancreatic cancer—A systemic review. Immunol. Lett..

[54] Bharti R., Dey G., Mandal M. (2016). Cancer development, chemoresistance, epithelial to mesenchymal transition and stem cells: A snapshot of IL-6 mediated involvement. Cancer Lett..

[55] Liang F., Ren C., Wang J., Wang S., Yang L., Han X., Chen Y., Tong G., Yang G. (2019). The crosstalk between STAT3 and p53/RAS signaling controls cancer cell metastasis and cisplatin resistance via the Slug/MAPK/PI3K/AKT-mediated regulation of EMT and autophagy. Oncogenesis.

[56] Zhao K., Lu Y., Chen Y., Cheng J., Zhang W. (2020). Dual inhibition of MAPK and JAK2/STAT3 pathways is critical for the treatment of braf mutant melanoma. Mol. Ther. Oncolytics.

[57] Duplomb L., Chaigne-Delalande B., Vusio P., Raher S., Jacques Y., Godard A., Blanchard F. (2003). Soluble mannose 6-phosphate/insulin-like growth factor II (IGF-II) receptor inhibits interleukin-6-type cytokine-dependent proliferation by neutralization of IGF-II. Endocrinology.

[58] Ge D., Gao A.C., Zhang Q., Liu S., Xue Y., You Z. (2012). LNCaP prostate cancer cells with autocrine interleukin-6 expression are resistant to IL-6-induced neuroendocrine differentiation due to increased expression of suppressors of cytokine signaling. Prostate.

[59] Kiu H., Nicholson S.E. (2012). Biology and significance of the JAK/STAT signalling pathways. Growth Factors.

[60] Martino N., Ramos R.B., Lu S., Leyden K., Tomaszek L., Sadhu S., Fredman G., Jaitovich A., Vincent P.A., Adam A.P. (2021). Endothelial SOCS3 maintains homeostasis and promotes survival in endotoxemic mice. JCI Insight.

[61] Fatih N., Camberlein E., Island M.L., Corlu A., Abgueguen E., Detivaud L., Leroyer P., Brissot P., Loreal O. (2010). Natural and synthetic STAT3 inhibitors reduce hepcidin expression in differentiated mouse hepatocytes expressing the active phosphorylated STAT3 form. J. Mol. Med..

[62] Stivala S., Codilupi T., Brkic S., Baerenwaldt A., Ghosh N., Hao-Shen H., Dirnhofer S., Dettmer M.S., Simillion C., Kaufmann B.A. (2019). Targeting compensatory MEK/ERK activation increases JAK inhibitor efficacy in myeloproliferative neoplasms. J. Clin. Investig..

[63] Bai Y., Wang W., Yin P., Gao J., Na L., Sun Y., Wang Z., Zhang Z., Zhao C. (2020). Ruxolitinib alleviates renal interstitial fibrosis in UUO mice. Int. J. Biol. Sci..

[64] Song X., Rahimnejad S., Zhou W., Cai L., Lu K. (2018). Molecular characterization of peroxisome proliferator-activated receptor-gamma coactivator-1alpha (PGC1alpha) and its role in mitochondrial biogenesis in blunt snout bream (*Megalobrama amblycephala*). Front. Physiol..

[65] Livak K.J., Schmittgen T.D. (2001). Analysis of relative gene expression data using real-time quantitative PCR and the 2^−ΔΔC_T_^ Method. Methods.

[66] Ding Z., Zhao X., Cui L., Sun Q., Zhang F., Wang J., Wang W., Liu H. (2019). Novel insights into the immune regulatory effects of ferritins from blunt snout bream, *Megalobrama amblycephala*. Fish Shellfish Immunol..

[67] Xu X., Tao L., Wang A., Li L., Fan K., Shen Y., Li J. (2020). Genome-wide identification of *JNK* and *p38* gene family in *Ctenopharyngodon idella* and their expression profiles in response to bacterial challenge. Comp. Biochem. Physiol. Part D Genom. Proteom..

[68] Li R., Li S., Chen Z., Jin Y., Li S., Li S., Bai Z. (2021). Grass carp (*Ctenopharyngodon idella*) stefin A: Systematic research on its cloning, expression, characterization and tissue distribution. Food Chem..

[69] Dai Y.S., Pei W.L., Wang Y.Y., Wang Z., Zhuo M.Q. (2021). Topology, tissue distribution, and transcriptional level of SLC34s in response to Pi and pH in grass carp *Ctenopharyngodon idella*. Fish Physiol. Biochem..

