# Supplementary material for: Classical Signaling and Trans-Signaling Pathways Stimulated by Megalobrama amblycephala IL-6 and IL-6R
Source: Int J Mol Sci. 2022 Feb 11;23(4):2019. doi: 10.3390/ijms23042019 (PMC8880141; doi:10.3390/ijms23042019)
Supplement: Supplementary file 1 [file ijms-23-02019-s001.zip › ijms-1588241-supplementary.pdf]

International Journal of
Molecular Sciences

MDPI

Article

Regulation of IL-6 classical signaling and trans-signaling in grass carp cells

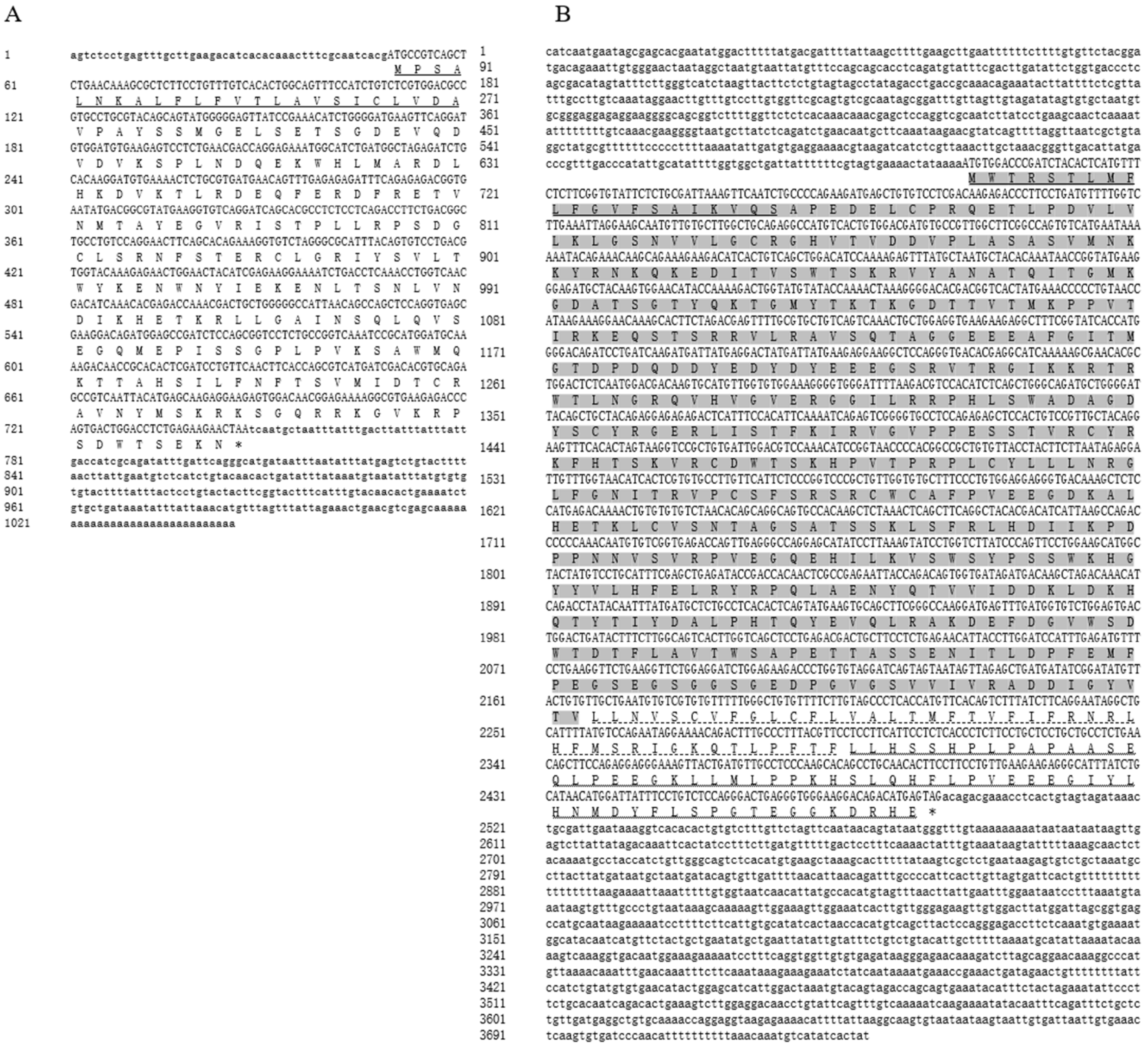

Figure S1. Complete CDS sequences of *mail-6* and *mail-6r*. UTRs are shown in lowercase. The deduced amino acid sequence is shown in the lower half of each row. The stop codon is displayed with a *. **(A)** The predicted maIL-6 signal peptide residues are underlined. **(B)** The maIL-6R outside amino acid sequence is marked with a prominent gray shadow, the transmembrane region sequence is underlined by dotted lines, and the inside sequence is underlined by wavy lines.

Table S1 Primers used in this study

Primers	Sequence (5′-3′)	Application	Reference
ciIL-6-F	CCGGAATTCATGGTGCCTGTGTACAGCAGA	plasmid	
ciIL-6-R	CCGCTCGAGGTTCTTCTCAGAGGTCCAGTCAC	construction	
maIL-6-F	CGCGGATCCATGGTGCCTGCGTACA	plasmid	
maIL-6-R	CCCAAGCTTGTTCTTCTCAGAGGTCCA	construction	
masIL-6R-F	CCGGAATTCGCCCCAGAAGATGAGCT	plasmid	
masIL-6R-R	CCCAAGCTTCACAGTAACATATCCGAT	construction	
18S-rRNA-F	ATTTCCGACACGGAGAGG	qPCR	
18S-rRNA-R	CATGGGTTTAGGATACGCTC		
IL-1β-F	TCTCCTCGTCTGCTGGGTGT	qPCR	Wang et al [40]
IL-1β-R	CAAGACCAGGTGAGGGGAAG		
IL-6-F	CTTGTTCAACTTCACCAGCG	qPCR	
IL-6-R	TAGTTCTTCTCAGAGGTCCAGTCA		
SOCS3a-F	GCTTTGAGAGGCTCCCCATAA	qPCR	
SOCS3a-R	GGTTTTGTAACGGTGAGACGG		
SOCS3b-F	GATTCCTGCTCCTTCTTCCT	qPCR	
SOCS3b-R	TCCGCCGTCTTCAACCTT		
Hamp-F	CCTTCAGACCGCAGCCGT	qPCR	
Hamp-R	GCCTTTGTTACGACAGCAGTTG		
ciIL-6R-F	CAGACCCCCCAACCAGAGT	fragment	
ciIL-6R-R	GAGAGGAATGAAGGAGCAACG	amplification	
ciGP130-F	CACCACTTACACCCTTTATGCT	fragment	
ciGP130-R	AACCTGCTACTGTTGACGCC	amplification	

Restriction endonuclease cut sites in primers are underlined.

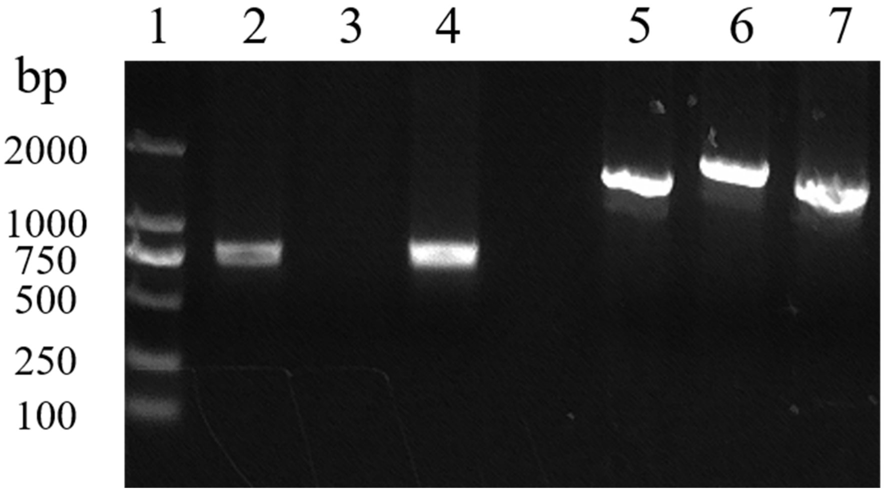

Figure S2 Expression analysis of *il-6r* and *gp130* in L8824 cells, CIK cells and primary hepatocytes. 1, Marker; 2, 3, 4 (*il-6r* in L8824 cells, CIK cells and primary hepatocytes); 5, 6, 7 (*gp130* in L8824 cells, CIK cells and primary hepatocytes).

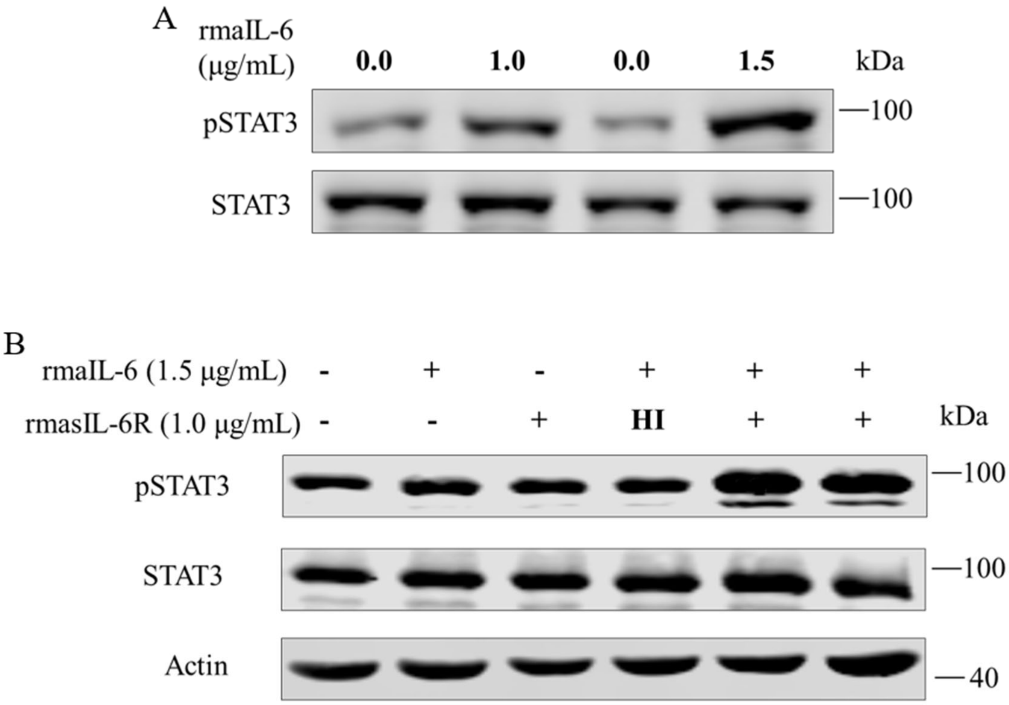

Figure S3. Phosphorylation of STAT3 in response to rmaIL-6 and rmasIL-6R. A, L8824 cells were treated with rmaIL-6 (1.0 and 1.5 μg/mL). B, CIK cells were treated with rmaIL-6 (1.5 μg/mL) and rmasIL-6R (1.0 μg/mL) alone or in combination. HI, heat inactivated protein.
